# The Relationship between Oral Squamous Cell Carcinoma and Human Papillomavirus: A Meta-Analysis of a Chinese Population (1994–2011)

**DOI:** 10.1371/journal.pone.0036294

**Published:** 2012-05-03

**Authors:** Changtai Zhu, Yang Ling, Chunlei Dong, Xifa Zhou, Feng Wang

**Affiliations:** 1 Department of Laboratory Medicine, Changzhou Tumor Hospital Soochow University, Changzhou, China; 2 Department of Oncology, Changzhou Tumor Hospital Soochow University, Changzhou, China; 3 Department of Radiation Oncology, Changzhou Tumor Hospital Soochow University, Changzhou, China; IPO, Inst Port Oncology, Portugal

## Abstract

**Background:**

Previous studies indicated that oral squamous cell carcinomas (OSCC) might be related to human papilloma virus (HPV) infection. However, up to now, there still lacks a large sample study to analyze the relationship between OSCC in a Chinese population and oral HPV infection. In the present study, we used a meta-analysis to evaluate the relationship of OSCC with HPV infection in a Chinese population.

**Methods:**

The reports on HPV and OSCC in a Chinese population published between January, 1994, and September, 2011 were retrieved via CNKI/WANFANG/OVID/MEDLINE databases. According to the inclusion criteria, we selected 18 eligible case-control studies. After testing the heterogeneity of the studies by the Cochran Q test, the meta-analyses for HPV and HPV16 were performed using the fixed effects model.

**Results:**

The overall positive rates of HPV and HPV16 in OSCC were 58.0% (354/610; 95% confidence interval [CI], 54.1–61.9) and 47.47% (169/356; 95% CI: 42.3–52.7), respectively; which were significantly higher than those in normal controls 10.44% (26/249; 95% CI: 7.2–14.7) and 7.1% (13/182; 95% CI: 4.2–11.8). Quantitative meta-analysis revealed that, compared with normal controls, the combined odds ratios of OSCC with HPV or HPV16 infection were 12.7 (95% CI: 8.0–20.0) and 9.0 (95% CI: 5.1–15.6), respectively. Both Begg's test and funnel plots revealed that no publication bias was found in this present study (*P*>0.05).

**Conclusions:**

High incidences of HPV infection (mainly involving HPV16) were found in the samples of Chinese OSCC. For the Chinese population, HPV infection elevates the risk of OSCC tumorigenesis. Prophylactic HPV-vaccination may reduce the burden of HPV-related OSCC in China.

## Introduction

As a member of the papillomavirus family of viruses, human papillomavirus (HPV) can infect humans by attacking the squamous cell of skin and mucous membranes. Based on the different nucleotide sequences, HPV can be divided into more than 200 genotypes by DNA sequencing, and 85 HPV genotypes have been well characterized [1], of which HPV16 and HPV18 as main high-risk types are more closely linked with malignant tumors [2]. In fact, HPV infection is a cause of nearly all cases of cervical cancer. Over 90% of all cervical cancers can be attributed to certain HPV types-HPV16 accounting for the largest proportion (roughly 50%) followed by HPV18 (12%), HPV 45 (8%), and HPV 31 (5%) [3]. Worldwide in 2002, an estimated 561,200 new cancer cases (5.2% of all new cancers) were attributable to HPV, suggesting that HPV is one of the most important infectious causes of cancer [4]. Cancers of the head and neck are usually caused by tobacco and alcohol, but recent studies show that about 25% of mouth and 35% of throat cancers in the United States of America are caused by HPV. Recent increases in incidence of oropharyngeal cancers in the USA have been attributed to HPV infection [5], [6]. In recent years, some studies by Chinese researchers have also focused on the relationship between oral squamous cell carcinoma (OSCC) and HPV oral infection. However, the differences of the odds rates were reported in different literatures. Therefore, it is necessary to implement a meta-analysis which aims to comprehensively evaluate the relationship between OSCC and HPV oral infection in a Chinese population.

## Methods

### Search Strategy

The keywords HPV, human papillomavirus, oral, oral cancer, head and neck cancer, tongue cancer, squamous cell carcinoma, oral carcinoma, buccal cancer, oral lesions, and Chinese population and China in address in Chinese language or in English language were used alone and in combination to search. The retrieved databases included China National Knowledge Infrastructure (CNKI)/Wanfang Database/OVID/MEDLINE. Finally, a total of 361 citations published between Jun, 1994 and Oct, 2011 were identified.

### Inclusion and Exclusion Criteria

**Figure 1:**
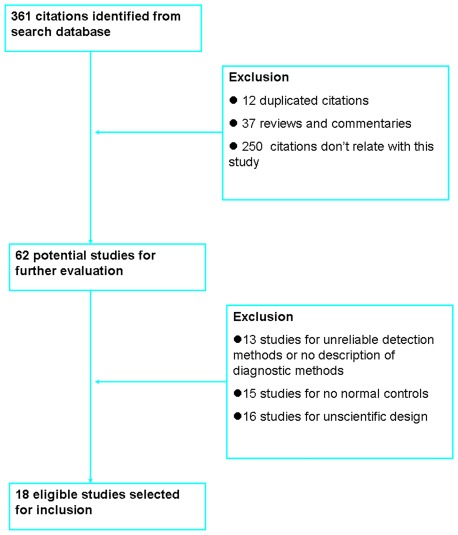
Flow diagram of the selection for the studies included in this meta-analysis.

The literatures included in the present study meets the following criteria: case-control studies; the participants belong to a Chinese population; a clear description of diagnostic methods was needed and the detection methods were reliable. The literatures excluded in this study were mainly due to the following reasons: lacks normal control group; reviews; the literatures include the repeated data; the research design isn't scientific and reasonable. Study identification and selection is elaborated in the flow diagram (Figure 1). Of 361 publications identified through an initial search of databases and conference abstracts, 343 were excluded for reasons explained in Figure 1. A total of 18 literatures met the eligibility criteria were included in this present study.

### Data Extraction

The data related to this study were extracted by two independent reviewers (ZCT and LY) based on a standardized data extraction program designed in advance. Any discrepancies were resolved by consensus or in consultation with a third reviewer (DCL). The data related to this study were shown in Table 1 and Table 2.

**Table 1:** Characteristics of studies investigating human papillomavirus (HPV) infection in oral squamous cell carcinoma (OSCC) and control samples.

	Year	Authors	Method	OSCC		Controls	
				Pos	Neg	Pos	Neg
1	1994	T Zhang et al	PCR	4	2	0	10
2	1996	YL Li et al	PCR	20	6	3	12
3	1996	L Lei et al	PCR	4	3	2	6
4	1998	WT Gao et al	PCR	30	10	4	36
5	1998	ZJ Tao et al	PCR	14	4	3	27
6	1998	WL Chen et al	PCR	11	10	3	18
7	2000	W Hou et al	Dot blot hybridization	16	16	0	10
8	2000	JQ Bu et al	PCR	29	21	0	5
9	2001	ZQ Lin et al	PCR	28	38	1	11
10	2004	C Yang et al	Hybridization in situ	14	16	0	20
11	2004	P Zhou et al	Hybridization in situ	17	16	0	8
12	2004	H Zheng et al	PCR	13	7	2	14
13	2005	LL Zhong et al	PCR	56	10	2	10
14	2006	QY Wei et al	Dot blot hybridization	19	18	4	17
15	2006	XF Tang et al	Hybridization in situ	20	10	0	5
16	2008	AE He et al	PCR	5	11	0	4
17	2011	LZ Li et al	PCR	24	32	1	11
18	2011	L Cheng et al	Hybridization in situ	30	26	1	9

Pos: Positive; Neg: Negative.

**Table 2:** Characteristics of studies investigating human papillomavirus (HPV) 16 infection in oral squamous cell carcinoma (OSCC) and control samples.

Year	Authors	Method	OSCC		Controls	
			Pos	Neg	Pos	Neg
1994	T Zhang et al	PCR	4	2	0	10
1996	L Lei et al	PCR	8	15	2	6
1998	WT Gao et al	PCR	29	11	1	39
1998	WL Chen et al	PCR	11	10	3	18
1998	ZJ Tao et al	PCR	12	6	0	30
2000	JQ Bu et al	PCR	23	27	0	5
2000	W Hou et al	dot blot hybridization	16	16	0	10
2001	ZQ Lin et al	PCR	28	38	1	11
2004	H Zheng et al	PCR	10	10	2	14
2006	QY Wei et al	dot blot hybridization	13	21	4	17
2006	XF Tang et al	PCR	10	20	0	5
2008	AE He et al	PCR	5	11	0	4

Pos: Positive; Neg: Negative.

### Data Analysis Data

The dichotomous data of HPV or HPV16 positive results in OSCC group and normal control group was summarized. OR and 95% confidence interval [CI] of OR were calculated for assessing the association between HPV or HPV16 infection and OSCC risk. The analysis of the heterogeneity of between-study was performed using the Chi-square-based Q test [7]. A *P* value less than 0.05 was considered significant for the heterogeneity. If no heterogeneity, a fixed-effect model was applied using the Mantel-Haenszel method [8]. Otherwise, the random-effect model with the DerSimonian-Laird method [8] was used. The potential publication bias was assessed graphically by Begg's test and analysis for funnel plots [9]. The statistical analyses were performed using RevMan 4.2 and Stata 10.0.

## Results

**Figure 2:**
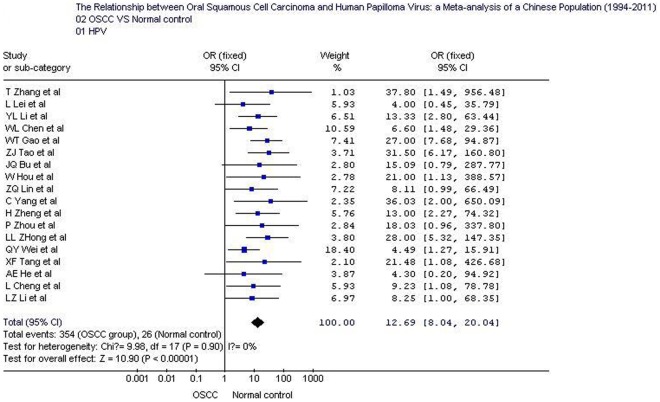
Forest plot of human papillomavirus prevalence in oral squamous cell carcinoma and control samples. OSCC: oral squamous cell carcinoma.

**Figure 3:**
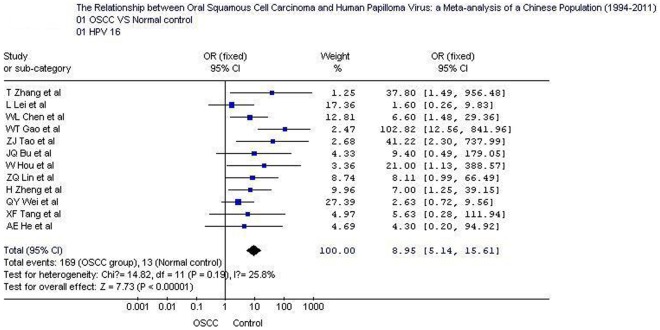
Forest plot of human papillomavirus 16 prevalence in oral squamous cell carcinoma and control samples. OSCC: oral squamous cell carcinoma.

**Figure 4:**
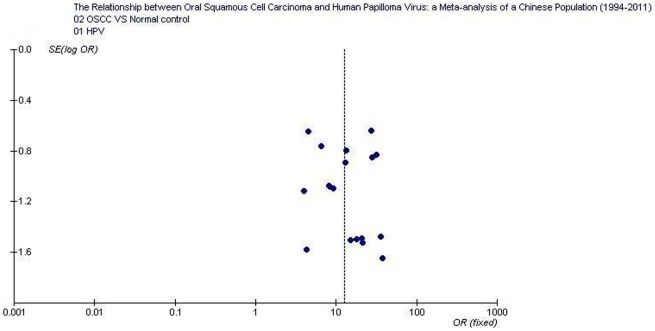
Funnel plot of the included studies of oral squamous cell carcinoma risk in HPV infection.

**Figure 5:**
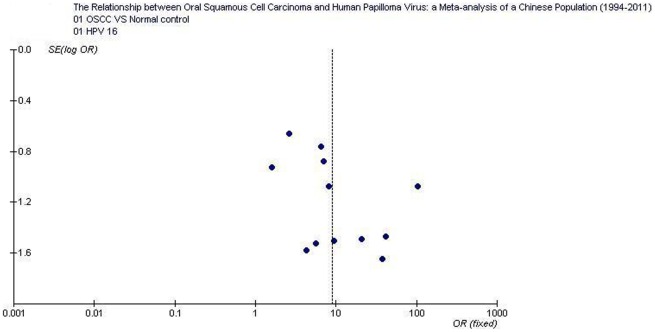
Funnel plot of the included studies of oral squamous cell carcinoma risk in HPV16 infection.

The overall positive rates of HPV and HPV16 in OSCC were 58.0% (354/610; 95% CI: 54.1–61.89) and 47.5% (169/356; 95% CI: 42.3–52.7), respectively; which were significantly higher than those in normal controls 10.4% (26/249; 95% CI: 7.2–14.9) and 7.1% (13/182; 95% CI: 4.2–11.8).Tests for the heterogeneity showed that, the Chi-square values for HPV and HPV16 were 9.98 and 14.82, respectively (*P*<0.05). Therefore, a fixed-effect model was applied. Quantitative meta-analyses showed that, compared with normal oral mucosa the combined odds ratio of OSCC with HPV and HPV16 infection were 12.7 (95% CI: 8.0–20.0) and 8.95 (95% CI: 5.1–15.6), respectively. The test for overall effect showed that the *P* value was less than 0.00001 (Z = 10.9). Forest plot analyses were seen in Figure 2 and Figure 3. Publication bias analysis using RevMan 4.2 software funnel plot of included studies were measured indicators of the graphics of the basic symmetry, all points is concentrated in the central funnel (Figure 4, 5). Begg's test (using Stata 10.0) showed that there had no publication bias (*P*>0.05).

## Discussion

Oral cancer belongs to head and neck cancer is any cancerous tissue growth located in the oral cavity. There are several types of oral cancers, 90% of which are OSCC and followed by glandular epithelial carcinoma (mucoepidermoid carcinoma, adenocarcinoma, adenoid cystic carcinoma, malignant pleomorphic adenoma, and acinar cell carcinoma) and undifferentiated carcinoma. Oral or mouth cancer most commonly involves the tongue. However, the tumorigenesis of OSCC is complicated. Smoking and other tobacco use are associated with about 75 percent of oral cancer cases [10]. Both alcohol use and chewing betel nut were also reported to be high-risk factors associated with tumorigenesis of oral cancer [11], [12], [13], [14], [15]. Patients after hematopoietic stem cell transplantation (HSCT) are at a higher risk for tumorigenesis of OSCC [16]. In addition, HPV, in particular type 16, have been found to be associated with oropharyngeal cancer [17], [18], [19], [20], [21]. The previous study confirmed that oral infection with HPV increased the risk of tumorigenesis of oropharyngeal cancer independent of tobacco use [22]. Sexually-transmitted HPVs also cause most of anal cancers and approximately 25% of oropharynx cancers [4]. Engaging in anal sex or oral sex with an HPV-infected partner may increase the risk of developing these types of cancers [17]. HPV is estimated to be the most common sexually transmitted infection in the United States [23]. Along with the elevation of HPV incidence, the incidence of oropharyngeal cancers in the United States had increased between 1973 and 2007, whereas that of cancers at other head and neck sites has decreased steadily [24]. The evidences revealed that the odds ratio of oral HPV infection increases with the number of recent oral sex partners or open-mouthed kissing partners [25] and nonsexual oral infection through salivary or cross transmission is also plausible [26].

There have some elucidated mechanisms of tumorigenesis of OSCC related to HPV infection. HPV viral sequences can integrate into the cellular DNA and the HPV “early" genes such as E6 and E7 can be expressed by host cells and then promote malignant transformation. In addition, E6 has a close relationship with the cellular E6-associated protein, which is involved in the degradation of p53 protein (a tumor suppressor factor can prevent cell growth and stimulates apoptosis in the presence of DNA damage), thereby cause tumors. In vitro studies suggested that the E6 protein of the HPV types implicated may inhibit apoptosis induced by ultraviolet light [27]. Many studies confirmed that HPV was closely associated with many tumors involving cervical cancer, as well as anal cancer, vulvar cancer, vaginal cancer, and penile cancer [4].

In 1983, OSCC was firstly reported to be associated with HPV infection [28]. Afterwards, many studies showed that a different degree of relationship might exist between OSCC and HPV infection. It is possible that the risk difference of OSCC with HPV infection varies from different regions and different populations. An alternative to HPV DNA testing is more specific novel biomarkers as HPV E6/E7 mRNA measuring the interaction of HPV with human cells [6], [29], [30], [31], [32]. In the study, we aimed to evaluate the relationship between OSCC and HPV infection in a Chinese population. Eighteen eligible case control studies showed that, HPV and HPV16 positive rates in normal oral mucosa ranged from 0–20% and 0–19.05%, respectively; while in OSCC were between 31.3%–84.9% and between 31.3%–72.5%, respectively. Based on the statistical results, the overall positive rates of detecting HPV and HPV16 in OSCC were 58.0% and 47.5%, respectively; which were significantly higher than those in normal controls 10.4% and 7.1%. Therefore, we believed that high infection incidences of HPV (mainly involving HPV16) were found in the samples of OSCC of a Chinese population. The recently published meta-analysis (1988–2007) [33] investigating HPV infection in OSCC and head and neck squamous cell carcinoma showed that the pooled prevalence of HPV DNA in the overall samples was 34.5%, and in OSCC was 38.1%. In comparison with the data, the overall incidences of HPV in this present meta-analysis were significantly higher (58.0% vs 38.1%, *P*<0.01).

The quantitative meta-analysis showed that, compared with normal oral mucosa, the pooled odds ratios of OSCC with HPV and HPV16 infection were 12.7 (95% CI: 8.0–20.0) and 9.0 (95% CI: 5.1–15.6), respectively. While another previous meta-analysis of the included literatures published in English-language journal between 1980 and 1998 revealed that [34], the likelihood of detecting HPV in normal oral mucosa (10.0%; 95% [CI], 6.1%–14.6%) was significantly less than that of OSCC (46.5%; 95% CI: 37.6%–55.5%), and the pooled odds ratio for the subset of studies directly comparing the prevalence of HPV in normal mucosa and OSCC was 5.4. Compared with the reported data, the pooled odds ratios of OSCC vs normal controls in this meta-analysis were higher significantly, suggesting that, for a Chinese population, HPV oral infection could cause a higher risk of tumorigenesis of OSCC.

The funnel plots of included studies showed that, the graphics is basically symmetric and all points is concentrated in the central funnel, indicating that no publication bias was found in this study. As a result, we believed that higher HPV infection incidences were found in the samples of OSCC of a Chinese population; HPV infection, mainly related to HPV16, elevates the risk of OSCC tumorigenesis. Therefore, HPV vaccines might also be a potential value in the prevention of OSCC. It is reported that, the HPV vaccines, which prevent infection with the HPV16/18 that cause 70% of cervical cancer, may lead to further decrease [35], [36]. Up to now, the definite effectiveness of the HPV vaccine in preventing OSCC has kept unknown.

However, the previous reports showed that the patients with HPV-positive oropharyngeal cancers had a lower risk of dying or recurrence than do those with HPV-negative cancers [37], [38], [39]. The majority of studies showed than HPV-associated OSCC were associated with a better prognosis than HPV-negative tumors in the majority of studies [40], [41], [42], [43], [44]. In the prior study, HPV-positive conferred a 60% to 80% reduction in risk of death from cancer compared with HPV-negative tumors [45]. So, screening for HPV in the patients with OSCC is also significant for assessing the prognosis of OSCC. Prophylactic HPV-vaccination may reduce the burden of HPV-related OSCC in China.

However, this meta-analysis may have two potential limitations. Firstly, most of the literatures included the present study didn't consider the potentially interfering factors, such as gender, age, socio-economic conditions, and lifestyle (smoking, chewing betel nut, and alcohol so on). Especially for lifestyle, some studies have shown that a close relationship was confirmed to be between OSCC and HPV infection. Besides, although OSCC group were diagnosed by pathological check, but lacked the subgroup analysis such as tumor location, clinical stage as well as degree of differentiation and so on. Considering that HPV infection plays an important role in tumorigenesis of OSCC, more scientific studies are worthwhile to do in the future.
